# Hospital and Institutional News

**Published:** 1912-02-10

**Authors:** 


### HOSPITAL AND INSTITUTIONAL NEWS.

### OUR REPORT AND ITS SEQUEL AT YORK.

Not only has our report on the York County Hospital done its work in attracting attention to certain conditions which required it, but also it has had an effect on the local papers which is both amusing and instructive. Thoroughly startled by the directness of our statements and not knowing in the least how to deal with them, the *Yorkshire Herald* seems to have decided that the best way out of its difficulty was to make the report unintelligible to its readers. So the editor has printed the report sandwiched between a few pious expressions, but has omitted necessary inverted commas, so that Sir Henry Burdett's remarks are inextricably confused into a hotchpotch seemingly the editor's own. What the editor of the *Herald* has done is to confuse a very direct and understandable report by turning it partly as quotation and partly as his own make up, practically rendering it impossible to distinguish between them. What Sir Henry really wrote will be found in The Hospital file. Our report either should be printed in full, or, if on the extract-sandwich system, with inverted commas, clearly to mark the line between our bread and butter and the York ham.

### THE CITY OF YORK ON TRIAL.

The gist of the whole matter at York is, that its citizens do not support the county hospital to anything like the extent other cities do theirs. The citizens of York are prosperous to an unusual degree; they have an Archbishop who is one of the ablest and most energetic of public men, and some of their manufacturers are amongst the richest and most prosperous in the country. Their hospital needs reconstruction, and the expenditure of a good round sum upon it. In similar circumstances other cities and towns have found the money promptly. Thus, Chester is spending £30,000 in consequence of Sir Henry's report on the Infirmary there. Is the City of York content to do less for its hospital and to let it remain an out-of-date, ancient, and uninteresting building, ill-suited to modern treatment and modern requirements? That is a question the people of the city and county of York must answer for themselves. It is their responsibility, and with them we leave it for the present.

### THE DOCTORS AND THE INSURANCE ACT.

Since we went to press last week the situation as between the medical profession and the Insurance Act has undergone a marked development. To begin with, all the medical corporations invited to confer with Insurance Commissioners declined the invitation, and all substantially on the same grounds. The Council of the College of Surgeons, in refusing the invitation, expressed the opinion that "the administration of medical benefits cannot be carried out under the Act with due regard to the interests of the public and the welfare of the medical profession, and that no satisfactory arrangement can be arrived at without an amending Act." For explicitness, directness, and dignity, it would be hard to improve on this summary of the case against the Act; and in the face of a series of refusals as decided as this, the Commissioners had no option but to "postpone" the conference to an unspecified date, which may be somewhere near the Greek kalends. It is thus made perfectly plain what the opinion of the profession really is, as expressed by its various colleges, and by such representative organisations as the British Medical Association. Moreover, the general public and the Press is also obtaining a clear insight into the reasons for this profound antagonism; and although the Lloyd Georgian Press fulminates threats of terrible vengeance, these are in reality but an indication of the consternation created among the Ministry by this revolt of the profession against the chicanery by which its interests were sacrificed in order to catch the votes of Friendly Society members.

### "THE PRACTITIONER" AGAIN ACTIVE.

Meanwhile it is worth noting that the energy of the editor of the *Practitioner* is unabated. Out of the 23,000 required pledges not to take service under the Act, 21,000 had been obtained at the end of last week, and the total was then still increasing. The laggards will, it may be hoped, be stimulated into action by a perusal of the counsel's opinions which have been secured by the *Practitioner* as to the prospects and possibilities of securing the "six point" *minimum* programme drawn up by the British Medical Association. The three counsel whose opinion was sought were Sir Edward Clarke, Mr. Danckwerts, and Mr. Stuart Bevan. Broadly speaking, their considered verdicts are the same; and they show that some of the six points are definitely excluded by the provisions of the Act, and that some others may be obtainable by grace of the Friendly Society representatives (who, if old experience is any guide, are about the last people in the world to be "friendly" to the doctors). These pronouncements from eminent lawyers merely serve to emphasise the magnitude of the betrayal by the Council of the British Medical Association of the interests entrusted to it by the members. Meanwhile the Council is shaping its course upon the lines of obtaining the six points by negotiation with the Insurance Commissioners and the Health Committees. That, at least, is the apparent meaning of the report which the Council caused to be published in last week's issue of the *British Medical Journal*. The simultaneous publication of the *Practitioner's* analysis of the three lawyers' verdicts completely knocks the bottom out of these absurd recommendations of the Council; and it must surely be dawning even on the members of that Council themselves that the criticisms of their conduct are not unjustified.

### A HOSPITAL PRESIDENT CAUGHT TRIPPING.

Very properly the Royal Infirmary at Halifax made its recent annual meeting an occasion for discussing the probable effect of the Insurance Act.

It was a great opportunity, unfortunately partly lost by the ill-considered action of the hospital's president, the Rev. G. E. Aspinall, J.P., who went out of his way to cast a most undeserved sneer at the resolutions passed by the British Hospitals Association at its London meeting in June, and at the Manchester Conference in September. These meetings made hospital opinion throughout the country; the British Hospitals Association has been, to quote Mr. Walter Alvey's opinion as published in The Hospital on the eve of the New Year, "the only body that has made any sustained effort to obtain for us recognition in the Bill." Once that opinion has been made, and the work done, it is easy for those who have merely reaped the reward for which the Association laboured to sneer at the perhaps not very exciting spade work which someone else had done. The ineptitude of Mr. Aspinall's complacent superiority over the value of the resolutions passed at London and in Manchester will be realised by all who appreciate the fact that if there had been no resolutions passed in London it would have been difficult to gather a representative muster in the first place, and in the second quite impossible to impress public opinion through the newspapers with the serious conviction that hospital managers had any policy at all. It is hardly credible that a hospital president who is also a clergyman should be so out of touch with public affairs as not to realise that the London Press will only print news, and that discussions, however brilliant, are not news, while resolutions in the concrete certainly are.

### THE REV. G. E. ASPINALL'S BAD MISTAKE.

This sort of ignorance in men who hold responsible positions makes one almost despair of public life. We had to remind the Chairman, in The Hospital of October 7, that even so eminent a hospital official as himself cannot be allowed to lead the weaker brethren astray without correction, though he also was impatient of the resolutions. Our reminder to him may as well be repeated to Mr. Aspinall, who has publicly fallen into the blunder which Sir William Cobbett managed to avoid. Once more let us repeat the commonsense of the matter as to the passing of these resolutions. "It was not to agree so much as to record in public a unanimous agreement for which the Manchester conference met. Without a resolution the decision of the conference would have been disregarded by the public and by the newspapers as the sounding of brass and the tinkling of cymbals. The real audience was not only that present in the room, it was the public outside it . . . Failing the passing of a resolution, to what could the members who attended point? . . . The vote of the conference has given to every hospital manager throughout the constituencies a weapon that was not even forged ten days ago." Mr. Aspinall's self-satisfied complacency to-day shows that these remarks were worth writing. He may not, on his own confession, "have got further than the excellent lunch." Others, fortunately for the voluntary hospitals, did so, with the result that "hospital benefit" is not yet a lost cause.

### A COTTAGE HOSPITAL AND THE INSURANCE ACT.

Like other institutions, cottage hospitals, particularly those in the growing districts around London, are debating how to deal with the difficult position created by the Insurance Act. So far as we know, the Acton Cottage Hospital is one of the first in the field with a definite plan of action. The district is largely an industrial one, and Mr. E. F. Hunt, the hon. secretary, fears to lose employers' subscriptions; so we understand that it is proposed, if not actually decided, to alter the hospital's regulations by admitting those patients who receive any benefit under the National Insurance Act, only upon payment of 5s. a week. Of course, accident cases will still be admitted free of charge, and all those who are too poor to pay, but it is felt that if persons are entitled to the benefits of the Act they are not too poor to pay. We understand, moreover, that the hospital's board believes that if hospitals generally would make such a regulation it would hasten the State's recognition of and contribution towards hospital benefit. What have other cottage hospital secretaries to say to this attitude? We should be glad to publish their opinions on the point.

### THE NORTHCOTE TRUST AND ST. THOMAS'S OUT-PATIENTS.

The Northcote Trust on Wednesday last week invited all the former sanatorium patients from the out-patients department of St. Thomas's Hospital to a social gathering. About sixty patients, some of whom had undergone open-air treatment as long as six years ago, attended. They wore buttonholes of different flowers to distinguish the various sanatoria, and had chiefly been at Malting, Frimley, Kelling, Peppard, and St. Catharine's, Ramsgate. With hardly one exception, we are informed, they looked extraordinarily strong and healthy, and were excellent testimonials to the supervision and home care that is devoted to every individual phthisical patient at St. Thomas's Hospital. At 8.30 Dr. J. J. Perkins gave an interesting lecture and limelight show. A vote of thanks was given to Dr. Perkins for so kindly coming, and hearty greetings to the Northcote Trust Phthisical visitor, Miss P. Hickling which showed clearly how warmly the guests appreciated her energy and enterprise. Every effort was taken to give an absolutely social and informal character to the At Home, and the Lady Almoner, Miss Cummins, only invited a few voluntary visitors, already friends of some of the patients, to join the party. A thoughtful observer could hardly have failed to realise the fact that invaluable as sanatorium treatment is in cases of early consumption, half its value is wasted unless the patients are carefully assisted to maintain their recovered strength and power of resistance.

### DOCTORS' SUCCESSFUL BOYCOTT.

The action of the Brighton medical men, as announced in The Hospital of January 20, in deciding to boycott the Workhouse Infirmary on the ground that the salary offered was insufficient, has proved so successful that the Guardians have decided to send a deputation to the Local Government Board to ascertain the official opinion. Meanwhile a special committee of investigation has been approved. This should come as a useful reminder of what medical solidarity may mean to those party papers which are busy attempting to ridicule the efficacy of a medical refusal to accept the duties proposed by an unamended Insurance Act.

### NEW PRESIDENT OF STROUD HOSPITAL.

The successor to the late president of Stroud General Hospital, Sir John Dorrington, Bart., has been elected in the person of Sir William H. Marling, Bart., on the proposition of Major Fisher, Chairman of the Hospital's Committee. The new president's first action was to animadvert on the Insurance Act, and he rather wittily remarked that "in July the heavy hand of the Government was going to be laid upon the angel of voluntary charity, and I fear her form may shrink under the touch." After remarking on the additional expenses to the hospital which the insurance of its staff would cause, a pleasant interruption was made by Sir Alfred Apperly, who promised to increase his annual subscription in order to meet this additional charge.

### THE NEW SURGICAL POST AT GUY'S.

In view of our comments on the recently established special departments at Guy's, in our issue of January 13, it is interesting to add that the post of surgeon in charge of the orthopædic department has been filled by the appointment of Mr. W. H. Trethowan, who qualified M.B., B.S., at London University in 1906, having obtained honours in three subjects and the gold medal for distinction in the whole examination. As we remarked previously, the holder of this appointment must "go abroad to familiarise himself thoroughly with Continental methods," and Mr. Trethowan, after studying Swedish drill, will act as chief clinical assistant under Mr. Rowlands in the orthopædic department, which finally will be given into his hands. The appointment, if one may judge from the hospital *Gazette*, is very popular, and there now seems every chance of orthopædics being given their due, which, if it has come late, promises now to be thoroughly conceded.

### A HOSPITAL AS A CINEMA THEATRE.

A legal question of some nicety arises in relation to the use of the cinematograph in hospitals. With a view to safeguarding the public against risk of fire, it was provided by the Cinematograph Act, 1909, that no exhibition for the purposes of which inflammable films are used shall be given elsewhere than in premises licensed for the purpose in accordance with regulations made by the Secretary of State. These regulations, which were issued in 1910, provided (*inter alia*) for adequate means of egress; sufficient fire appliances, and for the isolation of the cinematograph apparatus from the Auditorium. The Act and regulations do not, however, apply to "an exhibition given in a private dwelling-house to which the public are not admitted, whether on payment or otherwise." The question is—Does the hospital come within this exception? From the point of view of a man in the street, a hospital would probably not be regarded as a private dwelling-house; but when the object of the Act is considered, it is conceived that a hospital ought to be within the exception. The public are not admitted to a hospital in the sense that they are admitted to a "cinema" theatre; and when it is remembered that such an exhibition in a hospital would generally be given under the control and superintendence of scientific persons, the risk of accident is reduced to the vanishing-point. At the same time, there is a doubt about the matter which cannot fail to be very embarrassing to the management of institutions where moving pictures are used for educational purposes.

### A NICE LEGAL POINT.

The doubt is not altogether cleared up by a careful examination of the strict legal meaning of the words in the proviso above mentioned. What, for instance, is the legal meaning of the term "dwelling-house"? In the Housing of the Working Classes Act, 1890, it is defined to mean "any inhabited building." In relation to the crime of burglary it was thus defined in the Draft Criminal Code of 1879: "a permanent building, the whole or any part of which is kept by the owner or occupier for the residence therein of himself, his family or servants, or any of them, although it may at intervals be unoccupied." This definition, although it has not the force of statute, is based upon a long series of decisions, and if it were adopted for the purpose of construing the Cinematograph Act, it is submitted that a hospital would be an excepted building, unless the word "private" is of importance. The matron and the nurses, who are "servants of the owners" of a hospital, obviously reside in some part of the building. As to the use of the word "private," it might be argued that this was a mere redundancy, because any building to which the public are not admitted is private in one sense of the term.

### THE NEED FOR A TEST CASE.

We are informed that a letter from the Home Office, without committing the Department, contains the opinion that a hospital is not exempt. If this be really the case it is high time that the law was modified so as to admit of students being instructed by this means in every institution. On the other hand, if the semi-official declaration emanating from the Home Office be erroneous, it were well to obtain an authoritative legal ruling upon the matter. If a test case cannot be at once arranged, each institution which now uses the cinematograph should continue to use it, subject, as far as possible, to the State regulations. The authorities, then, if they think it worth while, will soon take the matter up.

### A DISGRACE TO IPSWICH.

The overcrowding at Ipswich Workhouse Infirmary, to which The Hospital has recently drawn attention, was the subject of consideration at a recent meeting of the Ipswich Guardians, presided over by Mr. W. H. Calver. The justice of the letter from the medical officer, Mr. J. R. Staddon, which we published on January 27, and which, we pointed out, proved that the responsibility for the present inexcusable overcrowding lay not with him but with the Guardians, is now manifest. The Clerk read an extract from the Local Government Board Inspector's report, in which Capt. G. Hervey said the "pressure had been so great that patients had had to be provided for on the floor. . . There were no infirm wards at the Ipswich Workhouse. . . . The day room . . . in which there would be space for forty-four (men) only, but 136 men used this room . . . the atmosphere was always more or less vitiated." A patient who went from the Infirmary to the Workhouse would thus fall out of the frying-pan into the fire, and vice versâ. As if to confess that no defence was possible, Mr. Calver suggested that the Workhouse Committee's report should then be read. "In regard to the overcrowded condition of the Infirmary," the Committee "beg to remind the Board that this matter has been considered by them on several occasions during recent years (dates then follow). On each occasion the proposals made by this Committee were rejected by the Board. The Committee is satisfied that the Workhouse and Infirmary are both seriously overcrowded, and the necessity for some extension to the buildings is greater than ever." Additional accommodation was the final recommendation of the report. Incredible as it may sound a certain Mr. Jackson suggested that a joint committee of the Workhouse Committee and of the Guardians should be formed, and, if you please, "report to the Board."

### HOW THE FARCE IS TO BE CONTINUED.

This undisguised burking of the situation reveals our Ipswich Bumbles at their worst; but the explanation of it is apparently supplied by a member of the Workhouse Committee, Mr. Boast, who unerringly put his finger on the motive of the backsliders by saying "the Workhouse Committee has put forward ideas which have been defeated time after time," and added, "Some people might try and gain popularity by keeping the rates down, but if it could not be done" the difficulty must be faced. It is now clear that the Ipswich Guardians have no shadow of a serious excuse for their neglect of this unchanging overcrowded condition, and local public opinion should, we hope, be not so degraded as to let them escape their responsibility by raising the trumpery cry of a rise in rates. In the end, of course, the joint committee was appointed to knock another nail in the Guardians' coffin by adding the last to this long list of disgraceful reports. We hope Mr. John Burns will step in and insist on additional accommodation.

### NEW MEDICAL STAFF OF THE COUNTY COUNCIL

At the end of last year the London County Council authorised the appointment of six full-time medical assistants in the Public Health Department, three to be on the permanent staff, each at the commencing salary of £400 a year, and the other three to be employed temporarily at the same salary, with the prospect of being placed on the permanent staff after one year's service. No fewer than 177 applications were received: such are the attractions of permanency and pensions in the public service. At a recent meeting the following appointments were made: Dr. J. G. Forbes, M.D.; Dr. F. C. Lewis, M.D.; and Miss A. A. Parson, M.B., were appointed to the permanent positions, and Miss J. L. D. Fairfield, M.D.; Miss J. R. F. Gilmour, M.B.; and Mr. A. J. Malcolm, M.R.C.S., to the temporary positions.

### TWENTY-SIX YEARS OF VOLUNTARY WORK.

So much interest was aroused by our article last week entitled "Two Useful Careers," with its graphic account of the life and records of a London as contrasted with a Provincial hospital secretary, that this week we propose to show that such records need not be confined to obituary notices alone. A third type of hospital worker is worth appreciation, the working man who gives for nothing all his spare hours and energies to hospital work. The Royal Bucks Hospital at Aylesbury provides an example in the person of Mr. Henry Nunn, Secretary of the Aylesbury and District Friendly Societies' annual collection fund. Mr. Nunn, who is still the moving spirit as he has been the founder of this movement, has given to this one work alone his evenings, and sometimes his Sundays, for three months in the autumn for about twenty-six years. These facts are telling, are they not? The spirit of personal service has found its expression and its happiness in these hours. In pounds and pence, too, the result has been that about £2,200 has been handed to the hospital in money, apart from many tons of vegetables, for certain of the members give their collections in kind.

### THE PUBLIC LIFE OF A WORKING MAN.

Comparing Mr. Henry Nunn's public usefulness with that of the two secretaries whose "useful careers" we described last week, a further striking parallel suggests itself. To all three, hospital work was only the starting point for social work of many kinds. It should always be so; it is so always when the worker is worthy of his work. To get back to the example: Mr. Henry Nunn, if, unlike the late Mr. Reade, he has not (so far as we know) been especially connected with work for the unemployed or as a charity trustee, advocated and was the actual proposer of the convalescent home benefit of the Hearts of Oak, now an enormous branch of the work of that Society. Further he assisted in organising the mass of detail necessary for the drafting of rules and the giving of information wherever medical branches of the Hearts of Oak were to be set up. The juvenile benefit of the same Society also was formed under his inspiration and care. Figures become fascinating in the light of a personality, and so we add here that the Juvenile Benefit Society has some 20,000 members to-day, has transferred about 60,000 to the adult society, and has paid away over £80,000 in sick and funeral claims.

### "A MAN WITH A MESSAGE"

All this, observe, the by-ways of hospital life, the radiations, as it were, of a man's enthusiasm for his local hospital. Such lives prove that the tireless appeals for money which are issued every year on behalf of our voluntary hospitals are, at least to the best among the men who issue them, in the nature of apostolic incitations. For every capable hospital secretary is a man with a message. Personal service is his mainspring. In the present case we have the fine example of a man with no message in that limited sense, but with a record of personal service that will inspire much emulation, and probably many messages, in others. Some suppose that such a record should be left untold till its author is beyond our praise: others that over twenty-five years of personal service demands, rather than deserves, a public account. The latter at least is our view, and we hope Mr. Nunn still has many years of work before him.

### A NEW ORGANISATION AT THE WEST END HOSPITAL.

Once again the immense value which an intelligently organised Ladies' Guild can add to its institution has been proved by the success achieved already through the recently organised Ladies' Guild at the West End Hospital for Diseases of the Nervous System. One of its first enterprises is its persuasion of the Hon. Ethel Cadogan to produce a children's play on the hospital's behalf. The play itself, called "The Woodland Princess," has been given at the Court Theatre, and it is hoped that over £100 will be cleared, thanks, in generous part, to the lady programme-sellers, who made £20 between them.

### SHIPS' SURGEONS AND DISPENSERS AT SEA.

The list of medicines and surgical stores which, by the Board of Trade regulations, must be carried by British vessels has long been obsolete; several of the items on the list were useless or comparatively seldom required, and very large numbers of drugs and instruments which are in these days indispensable were not included at all. It is therefore to be noted with satisfaction that the Board of Trade has approved the recommendations of a Special Committee appointed to consider and advise upon this question, and the new lists of medical and surgical stores come into force immediately. The reforms contemplated fortunately include even more urgent and valuable alterations than those alluded to. Thus in the case of emigrant ships whose complement of passengers and crew exceeds 1500, the Board recommends, though it does not directly order, that a second medical officer should be carried, and that a qualified dispenser should also be appointed. These excellent suggestions are so obviously the wise and humane course that it is almost a pity they are not to be enforced as regulations.

### THE MEN RESPONSIBLE FOR THE REFORMS.

The composition of the Committee by which the scales were drawn up is a guarantee that proper provision has been made for emergencies. The Committee was composed as follows: Dr. Nestor Tirard, joint editor of the British Pharmacopœia, nominated by the Royal College of Physicians; Mr. Clinton T. Dent (Royal College of Surgeons), Mr. A. J. Phillips (Pharmaceutical Society), Dr. W. H. Broad (The North Atlantic Steamship Passenger Line), Dr. C. Burland, and Dr. E. W. S. Evans (Board of Trade).

### CREW HOSPITALS FOR CARGO VESSELS.

The Board of Trade strongly approves the recommendation of the Committee concerning "the absence on board cargo vessels of any separate accommodation for members of the crew who fall ill or meet with accidents. We are of opinion that it would be very desirable that some suitable provision should be made on board all ocean-going cargo vessels for the separate and reserved accommodation of sick persons, and trust the Board will see fit to use its influence with shipowners in this direction." Such reserved space is entitled to be certified as the "crew's hospital" and to be included in the deductions allowed from the gross tonnage of the ship. By the way, a new edition of the "Ship Captain's Medical Guide" will be issued by the Board shortly.

### £80,000 FOR LEICESTER INFIRMARY.

We are pleased to announce a series of very generous bequests to the Leicester Infirmary. The principal one arises under the will of the late Mr. Alfred Adderly, J.P., of Woodhouse Grange, Leicester, whose will has recently been proved at upwards of £108,000. Legacies apart, Mr. Adderly bequeaths the whole of his estate to his wife for life, upon her death to his daughter for life, and, subject thereto, the whole upon trust for the Leicester General Infirmary. It is estimated that the Infirmary may eventually receive a sum of about £80,000 under this will. Mr. Adderly, it is worth adding, was a Life Governor of the Infirmary and contributed to the fund for its reconstruction. His name now will be associated, we have no doubt, in some prominent manner with the institution to which he gave so much.

### THE SALE OF POISONS.

During the past week or so some curious statements concerning the sale of poisons have found their way into London papers. For instance, the *Morning Post* informed its readers that pharmacists were desirous that cocaine should be scheduled as a poison and that ammonia if sold at a chemist's shop must be handed over the counter by a duly qualified pharmacist. Another morning paper was guilty of the statement that sulphonal was included in Part I. of the Schedule to the Pharmacy Acts. These are only examples of quite a number of mistakes made by writers in the lay Press within a few days. Cocaine, of course, has been scheduled as a poison for many years, and sulphonal is in Part II. of the Schedule which makes its sale subject to conditions totally different from the sale of poisons in Part I. The mistake regarding the sale of ammonia no doubt rose out of a misapprehension of the new regulations; so long as ammonia is sold in a bottle distinguishable by touch from ordinary bottles and the bottles are labelled with the words "Poisonous—Not to be taken," together with the name and address of the seller, it may be sold by anyone as heretofore.

### THE PHARMACEUTICAL SOCIETY AND THE PRIVY COUNCIL.

No doubt the subject of poisons is considered topical because it is understood that the Council of the Pharmaceutical Society intends to ask the Privy Council to add to the Poison Schedule certain drugs similar in effect to sulphonal. It is therefore worth while to add that before a substance becomes a poison in the legal sense it must be in the Schedule of Poisons to the Poisons and Pharmacy Act, 1908, and before a substance can be added to the Schedule the Council of the Pharmaceutical Society has to pass a resolution that such a substance should be added, and this resolution is submitted to the Privy Council for approval; if the resolution be confirmed the substance becomes subject to the restrictions as to the sale of scheduled poisons. A scheduled poison may only be sold by retail by a duly registered pharmacist, who here again must take the package with the name of the substance, the word "Poison" and the name and address of the seller. But the sale of substances in Part I. is subject to further restrictions; they may be sold only to persons known to, or introduced to, the seller, and the purchaser is required to append his signature to a record of the transaction in the "Poison-book." There can be no doubt that a number of drugs which at present are sold without restriction should, in the public interest, be added to the Schedule.

### HOSPITAL SUNDAY FAILURE: THE REMEDY.

In spite of the recent attempt to revive Hospital Sunday in Sheffield, it has proved a miserable failure again. The total amount collected is under £2,000. Even locally it is admitted that "this year's figure ranks among the lowest on record." A feat, by the way, not difficult in view of the fact that £2,134 is quoted as the highest figure for twenty years. The fault, of course, is with the Sheffield clergy, for whom no criticism can be too severe. With them, and with them alone, rests the power to make Hospital Sunday a success. Complete indifference and gross inertia are the only possible corollaries to be drawn from the hopeless fiasco of Sheffield's "effort" this year. We should like to see the Sheffield hospitals—why not under the leadership of such a man as Mr. Philip K. Wake, the Chairman of the Royal Hospital?—indulge in a little courageous plain speaking and charge the local clergy of all denominations with the dereliction of duty that is certainly theirs. If first the hospital chaplains were made to show an account of their stewardship—to report, in fact, exactly what endeavour, if any, they had made—a useful precedent would be created. The bringing to book of the chaplains would probably be quite enough to make the rest of the clergy wake up to their responsibilities. Why should the hospital chaplain be the only hospital official who is allowed to escape a personal explanation before the committee for slackness in his work? To attract the support of church-people or chapel-goers is certainly as much the chaplain's duty outside, as the visiting of the sick inside, the wards.

### SHOULD HOSPITAL CHRISTMAS BE ABOLISHED?

The Editor of the *St. Bartholomew's Hospital Journal*, which is a lively number this month, publishes extracts from an anonymous correspondent who says he is "sick of this canting humbug about Christmas in the hospital. For the benefit of the staff, both medical and nursing, desirable patients are kept in for weeks or even months, long after any necessity of treatment has departed. They are pampered and petted whilst the really needy and sick poor are crowded out." This is, of course, an old croaker who makes his appearance about every three years.

### AN OLD CROAKER'S RE-APPEARANCE.

Not only has the Editor of the *Journal* recognised that now, when the festivities are over though the memory of them is still fresh, is just the time to raise the question, but he prints the following comment of his own: "Why cannot something be done for the poorest of the out-patients, for the wholly destitute? One hospital we know holds annually its 'out-patient tea,' and it is not asking too much to hope that we may in future achieve something similar." There is, no doubt, a good deal of sense in this as far as it goes, but it leaves the main question unanswered. That question is whether it is preferable to deprive a few semi-convalescent patients of what has become in fact a traditional treat, or whether everything, not making Christmas any exception, should give way to waiting cases? It is not so easy to answer as it sounds. Perhaps our readers will tell us.

### THIS WEEK'S DRUG MARKET.

It is not possible to form a reliable opinion at present as to the future of the market for cod-liver oil. Buyers are holding back for the present, and this is perhaps the best policy to adopt. There has been another advance in the price of santonin, a drug which was thought to have reached its maximum price when it was at least 25 per cent. below its present figure; and as the Russian-Turkestan Syndicate has a monopoly the price may be raised until it reaches a figure high enough to cause a decrease in the demand; the small crop of raw material accounts to some extent for the advance, but not entirely. It is not unlikely that rhubarb will advance steadily in price owing to the troubles in China. Arnica is scarce and tends dearer, and the same remarks apply to gentian root. A good business has been done in buchu leaves at good prices. Menthol tends rather lower in price. Balsam tolu is now in more plentiful supply, and the price is a trifle lower. Balsam copaiba still remains scarce, and prices tend upwards. Opium is unchanged, and buyers are holding off as long as possible; should there be any noticeable demand, it is quite probable that the value would advance. Oil of star aniseed still shows a tendency to advance in price. The sharp frost which has just been experienced increased the demand for such articles as eucalyptus oil, quinine, and other drugs, but the cold spell was of too short a duration to cause any material diminution of the supply.

